# La Autoantigen Induces Ribosome Binding Protein 1 (RRBP1) Expression through Internal Ribosome Entry Site (IRES)-Mediated Translation during Cellular Stress Condition

**DOI:** 10.3390/ijms17071174

**Published:** 2016-07-20

**Authors:** Wenqing Gao, Qi Li, Ruiyu Zhu, Jian Jin

**Affiliations:** Laboratory of Molecular Pharmacology, School of Pharmaceutical Sciences, Jiangnan University, 1800 Lihu Road, Wuxi 214122, China; gwqjsks@163.com (W.G.); liqijswx@163.com (Q.L.)

**Keywords:** ribosome binding protein 1 internal ribosome entry site (RRBP1 IRES), paclitaxel (PTX), adriamycin (ADM), serum-starvation, La autoantigen (La)

## Abstract

The function of ribosome binding protein 1 (RRBP1) is regulating the transportation and secretion of some intracellular proteins in mammalian cells. Transcription of RRBP1 is induced by various cytokines. However, few studies focused on the process of RRPB1 mRNA translation. The RRBP1 mRNA has a long 5′ untranslated region that potentially formed a stable secondary structure. In this study, we show that the 5′ UTR of RRBP1 mRNA contains an internal ribosome entry site (IRES). Moreover, the RRBP1 expression is induced by chemotherapeutic drug paclitaxel or adriamycin in human hepatocellular carcinoma cells and accompanied with the increased expression of La autoantigen (La), which binds to RRBP1 IRES element and facilitates translation initiation. Interestingly, we found IRES-mediated RRBP1 translation is also activated during serum-starvation condition which can induce cytoplasmic localization of La. After mapping the entire RRBP1 5′ UTR, we determine the core IRES activity is located between nt-237 and -58. Furthermore, two apical GARR loops within the functional RRBP1 IRES elements may be important for La binding. These results strongly suggest an important role for IRES-dependent translation of RRBP1 mRNA in hepatocellular carcinoma cells during cellular stress conditions.

## 1. Introduction

In eukaryotic cells, expression level of proteins is determined and regulated by mRNA translation according to a ribosome scanning mechanism [1]. In this mechanism, the 40S ribosomal subunit conjugating with various initiation factors recognizes the methyl cap structure on the 5′ end of a mRNA and scans the transcript until encountering the initiator methionine codon in the proper context for translation [2]. In addition to ribosome scanning, 40S subunits enter mRNA molecules in internal ribosome entry sites (IRES), which have hundreds of nucleotides located downstream of the capped 5′ ends of mRNAs. IRESs were firstly found in picornaviuses and then a number of eukaryotic mRNAs, including NF-κB repressing factor (NRF) [3], fibroblast growth factor 2 protein (FGF2) [4], the protooncogene c-myc [5,6], vascular endothelial growth factor (VEGF) [7,8], and X-linked inhibitor of apoptosis protein (XIAP) [9], were identified as IRESs. The function of IRES was retaining or enhancing the expression of its regulated protein, when cap-dependent translation was downregulated under pathophysiological conditions, such as apoptosis, mitosis, genotoxic stress, and heat shock [10]. Therefore, IRES-mediated translation was important for the survival of several tumor cells [11]. However, the mechanism of IRES-mediated translation was not clear. Several studies revealed that trans-acting factors (ITAFs) were necessary for the activity of cellular and viral IRESs [12,13,14]. For instance, La autoantigen (La) and polyprymidine tract binding protein 1 (PTBP1) are two important ITAFs, which conjugated to the IRES region of mRNA and induced the conformational change to facilitate the recruitment of the ribosome for IRES [15,16,17,18]. 

Ribosome binding protein 1 (RRBP1) is an endoplasmic reticulum membrane protein that is essential for the ribosome binding and the translocation of nascent proteins across the membrane of the rough endoplasmic reticulum. Recently, some researchers reported the increase expression of RRBP1 protein in some tumor cells, indicated the great role of RRBP1 for survival, malignancy maintenance, and adaptation to ER stress in tumor cells. In these reports, the enhancement of RRBP1 protein expression in tumor cell is attributed to the increase RRBP1 mRNA caused by gene amplification and/or the raise of transcription [19,20]. However, the regulation of RRBP1 expression at the translational level was ignored. In searching the RRBP1 gene, we found RRBP1 mRNA contains an unusually long 5′ UTR. Thus, RRBP1 5′ UTR potentially formed stable secondary structures and may directly recruit the ribosome subunit and initiate translation. 

Here, 5′ UTR of RRBP1 mRNA was inserted into two different bicistronic reporter vectors to detect its IRES activity in cells. In addition, RRBP1 protein expression was tested in human hepatocellular carcinoma cells following treatment with inducers of cell stress and La may contribute to regulate RRBP1 IRES-mediated translation initiation during cellular stress conditions. This study was beneficial to gain a better understanding of the IRES-mediated RRBP1 translation mechanism and provide an effective method for cancer therapy.

## 2. Results

### 2.1. Enhanced Protein Synthesis Contributes to Overexpression of RRBP1 during Stress Conditions

**Figure 1:**
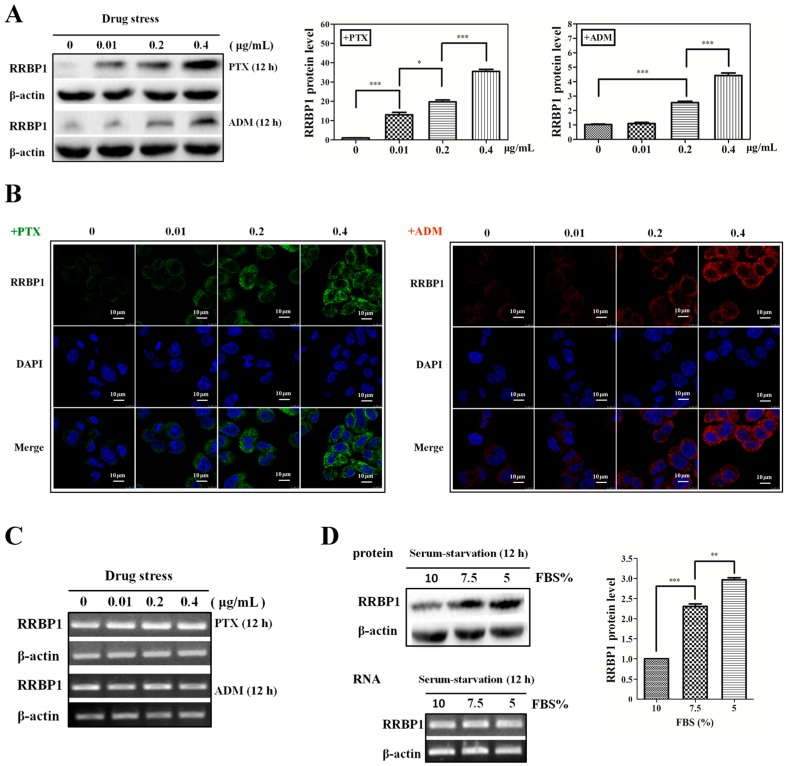
Enhanced protein synthesis contributes to overexpression of ribosome binding protein 1 (RRBP1) during stress conditions. (**A**) Human hepatocellular carcinoma cells (Bel7402 cells) were treated with paclitaxel (PTX) or adriamycin (ADM) at different doses (0.01, 0.2, 0.4 μg/mL) respectively, for 12 h. The protein expression of RRBP1 was examined via Western blot analysis and normalized to β-actin. The RRBP1/β-actin ratio from treated cells was compared with the ratio from untreated cells, which is set to 1 (*: *p* < 0.05; ***: *p* < 0.0005); (**B**) Immunofluorescence showed dose-dependent cytoplasm expression of RRBP1 in Bel7402 cells treated with PTX or ADM; (**C**) mRNA expression levels of RRBP1 after treatment with increasing concentrations of PTX or ADM in Bel7402 cells. mRNA levels were determined with reverse transcription-PCR (RT-PCR); and (**D**) the expression of RRBP1 protein was increased during serum starvation in Bel7402 cells. RT-PCR and Western blotting showed different levels of transcription and translation of RRBP1 in Bel7402 cells growing at serum-starvation and normal conditions (**: *p* < 0.005; ***: *p* < 0.0005).

Previous studies have shown that RRBP1 is a key player in the initial maintenance of cancer cell survival during conditions such as cellular stress and apoptosis [19,21]. To investigate whether the expression of RRBP1 was affected during cellular stress, we measured the endogenous protein and mRNA levels of RRBP1 in human hepatocellular carcinoma cells (Bel7402) treated with paclitaxel (PTX) and adriamycin (ADM), which are applied as chemotherapy drugs in cancer. In Figure 1A, the RRBP1 protein expression level was greatly raised with the increase of PTX from 0.01 to 0.4 μg/mL and ADM from 0.2 to 0.4 µg/mL. The RRBP1 expression under PTX was 7–12 folds higher than the same concentration of ADM, indicating PTX was a more effective pressure than ADM. In addition, immunocytochemical analyses showed that intense RRBP1 expression in the cytoplasm but weak expression in the nucleus of Bel7402 cells (Figure 1B). Drug treatment, especially PTX, significantly increased the expression of RRBP1 protein in cytoplasm and this expression was does dependent. Recently, some studies have reported that RRBP1 can also interact with microtubules and mediate interactions between the endoplasmic reticulum and microtubules. Overexpression of RRBP1 in mammalian cells may induce an elongated morphology and enhanced acetylated microtubules [22]. Similarly, we also found the highly expression of RRBP1 induced by 0.4 µg/mL PTX resulted in an elongated morphology in Bel7402 cells compared to the untreated cells. As a more effective drug, PTX targets tubulin, stabilizes the microtubule polymer, and protects it from disassembly, may cause upregulation of RRBP1 in Bel7402 cells. To determine the mechanism for the increase of RRBP1 protein expression, the transcription level was analyzed by reverse-transcription PCR (Figure 1C). The results show that the mRNA of RRBP1 was maintained at a constant level after treating with different concentrations of PTX and ADM.

It is known that with limited nutrient availability may lead cells to apoptosis, such as serum starvation. We found that RRBP1 protein expression in Bel7402 cells was also induced by serum starvation without an obvious change in mRNA level (Figure 1D). RRBP1 expression was 2.3-fold and 2.9-fold higher in cells cultured with 7.5% and 5% FBS, respectively, than that in cells growing at normal medium (10% FBS). These results indicate that the increased RRBP1 protein expression in Bel7402 cells during stress condition is not due to enhanced transcription of the RRBP1 mRNA, but the result of increased protein synthesis.

### 2.2. Analysis of IRES Activity within the 5′ UTR of RRBP1 mRNA

**Figure 2:**
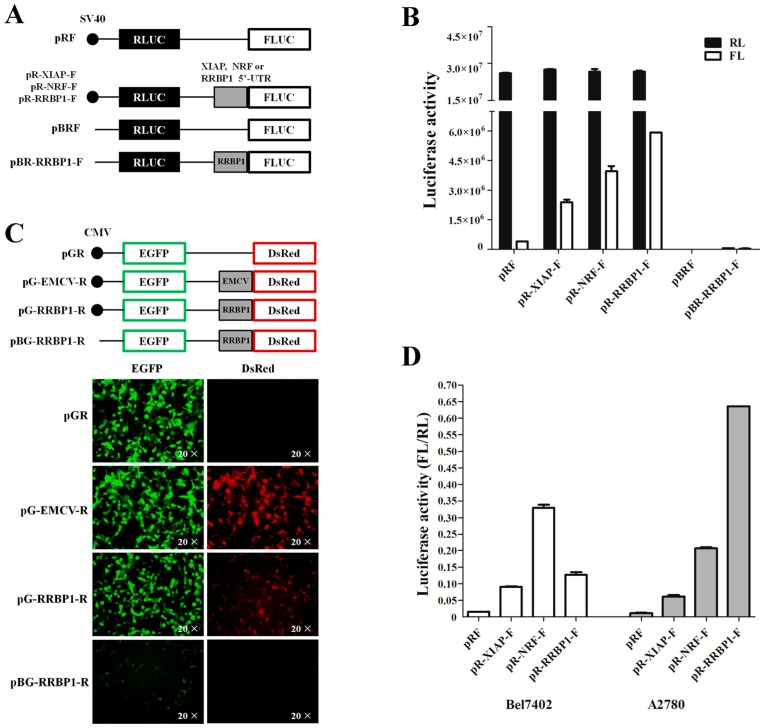
Analysis of internal ribosome entry site (IRES) activity of the RRBP1 5′ UTR sequence. (**A**) Dual-luciferase reporter vector construction. Positive IRES activity sequence, X-linked inhibitor of apoptosis protein (XIAP) and NF-κB repressing factor (NRF), and detected RRBP1 5′ UTR sequences were inserted between Renilla and firefly luciferase in plasmid pRF with or without promoter simian virus (SV40); (**B**) both luciferase activities were determined following transfection in human embryonic kidney 293 (HEK293) cells; (**C**) dual-fluorescent protein reporter vector construction. Positive viral IRES activity sequence, encephalomyocarditis virus (EMCV), and detected RRBP1 5′ UTR sequence were inserted between red and green fluorescent protein genes with or without promoter cytomegalovirus (CMV). The indicated plasmid was then transfected into HEK293 cells and the expression efficiency of the reporter genes were analyzed by fluorescence microscopy; (**D**) dual-luciferase reporter vector was transfected into Bel7402 and A2780 cell lines and luciferase activities from each cell line was measured, respectively. The ratio of firefly luciferase (FL) to renilla luciferase (RL) represented IRES activity of the inserted 5′ UTR. The expression data are presented as the mean ± SEM (standard error of the mean) of triplicate samples.

The 5′ UTR of RRBP1 is a long and GC-rich sequence (277 bases, NM_004587.2), indicating that it may be translated in a cap-independent manner. To determine if the RRBP1 5′ UTR has an IRES, the synthesis corresponding DNA (RRBP1 5′ UTR) and control sequences (XIAP and NRF 5′ UTRs) were inserted into the bicistronic reporter vector pRF between Renilla and firefly luciferases (Figure 2A). The Renilla luciferase (RL) is translated from the first cistron by a cap-dependent mechanism. However, the firefly luciferase (FL) is only translated when the inserted sequence possessed IRES activity. Therefore, the ratio of FL to RL is a measure of IRES activity of any inserted sequence.

The generated vectors were transfected into human embryonic kidney 293 (HEK293) cells and the luciferase data were determined. While the relative RL activity showed no difference among the pRF constructs and the 5′ UTR-containing pRF constructs, a significant elevation of relative FL activity was detected in the 5′ UTR-containing pRF constructs (Figure 2B). It has been reported that XIAP and NRF were well-characterized strong IRES elements in different cell lines [23]. After comparing with these positive controls, we found that the IRES activity of RRBP1 was higher than XIAP and NRF in HEK293 cells, suggesting that there was a more effective IRES element in RRBP1 RNA. To exclude the expression of firefly luciferase induced by a cryptic promoter in RRBP1 5′ UTR, the simian virus 40 (SV40) promoter deleted vector pBR-RRBP1-F was constructed and transfected into HEK293 cells (Figure 2A). The RL and FL activity was analyzed and no luciferase activity was detected (Figure 2B). This result indicates that no cryptic promoter existed in RRBP1 sequence.

Previous studies have demonstrated that poliovirus works when inserted into pRF, but not when the order of reporter genes is reversed [24]. The c-myc sequence exhibited strong IRES activity when inserted into pRF but not when tested with a βgal/CAT vector, suggesting that the IRES activity may dependent on the choice of reporter genes [6]. The human sensitivity to nitrogen mustard (hSNM1) 5′ UTR was tested with a dual-fluorescent protein reporter vector (pDsRed-EGFP) [25]. However, we found that there was obvious expression of EGFP in HEK293 cells transfected with pDsRed-EGFP vector (data not shown). To further verify that RRBP1 5′ UTR contains an IRES we inserted its sequence into a second bicistronic reporter vector pEGFP-DsRed (pGR), in which the fluorescent protein reporter genes are reversed. Moreover, insertion of a known viral IRES, encephalomyocarditis virus (EMCV), between the two cistrons is a strong positive control. As shown in Figure 2C, the presence of the RRBP1 5′ UTR remarkable simulated the expression of DsRed, but had no effect on the upstream cistron in HEK293 cells. We also deleted the cytomegalovirus (CMV) promoter from the pG-RRBP1-R vector to exclude the possibility of a cryptic promoter in the IRES element, and then transfected the resulting vector into HEK293 cells and no expression of DsRed protein was detected. Taken together, the above data strongly confirmed that the 5′ UTR of the RRBP1 mRNA had an IRES element. 

To investigate the activity of RRBP1 IRES in cancer cells, we transfected the IRES-containing pRF constructs into Bel7402 and A2780 cell lines and the relative luciferase activities were assayed, respectively. IRES activities of XIAP, NRF, and RRBP1 in two cancer cell lines exhibited completely differently (Figure 2D). A 54-fold increase in the FL per RL ratio was detected from the RRBP1 IRES-containing vector in A2780 cells, in contrast with a nine-fold increase observed in Bel7402 cells in comparison with that from the pRF vector. Moreover, the activity of RRBP1 IRES was greatly higher than XIAP especially in A2870 cell line (10.3-fold). The IRES activity difference of RRBP1 and positive controls may be caused by the expression of trans-acting factors in various cell lines. 

### 2.3. The RRBP1 IRES Upregulates Translation during Stress Conditions

**Figure 3:**
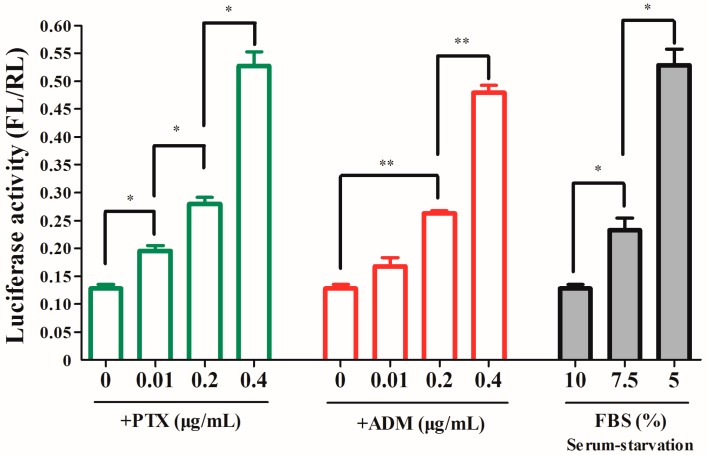
The RRBP1 IRES upregulates translation during stress conditions. The construct pR-RRBP1-F shown in Figure 2A was transfected into Bel7402 cells treated with and without increasing concentration (0.01, 0.2 and 0.4 μg/mL) of PTX or ADM, respectively. Meanwhile, the vector was also transfected into Bel7402 cells growing under normal medium and serum-starvation conditions. FL and RL activity were determined and the IRES activity was shown. The expression data are presented as the mean ± SEM of triplicate samples (*: *p* < 0.05; **: *p* < 0.005).

It is known that IRES-mediated protein translation is initiated during cellular stress and apoptosis. To determine whether elevated IRES activity increases RRBP1 protein synthesis during stress conditions, Bel7402 cells were transfected with the pR-RRBP1-F vector and then subjected to drug treatment or serum starvation. We observed that the IRES activity was increased gradually in cells when treated with PTX from 0.01 to 0.4 μg/mL or ADM from 0.2 to 0.4 μg/mL (Figure 3), which was similar to the enhancement of RRBP1 protein expression level (Figure 1A). We also found that the IRES activity of RRBP1 was enhanced 1.8–4.1-fold in Bel7402 cells during serum-starved condition. These results show a positive correlation between RRBP1 translational efficiency and RRBP1 IRES activity. Therefore, the IRES activity of RRBP1 5′ UTR was the main reason for the rise of RRBP1 protein expression during stress conditions.

### 2.4. La Interacts with and Enhances the RRBP1 IRES under Drug Pressures

**Figure 4:**
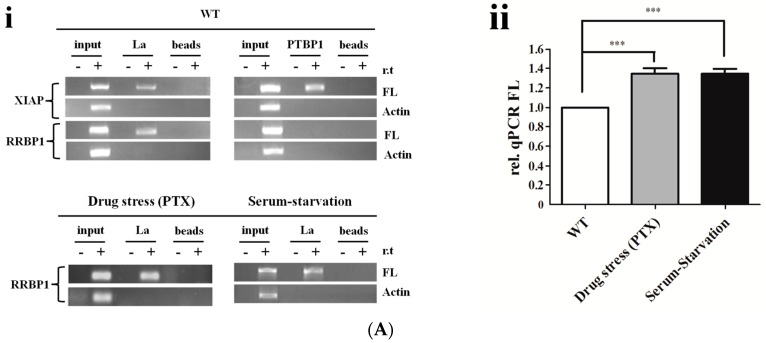

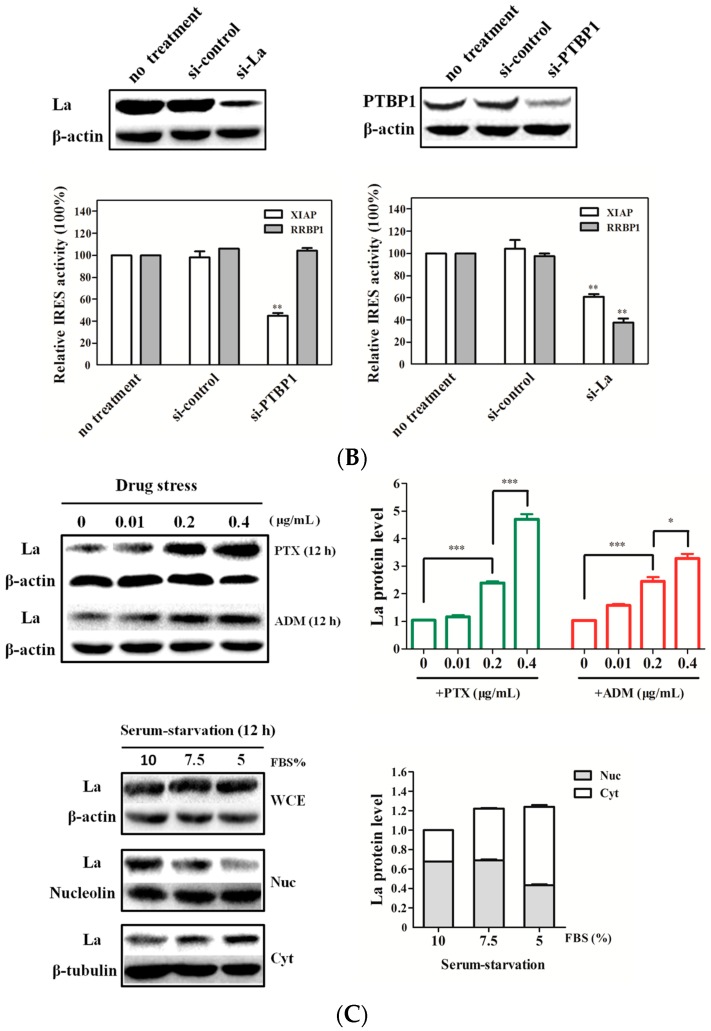
La interacted with RRBP1 5′ UTR sequence and enhanced the RRBP1 IRES activity in Bel7402 cells under drug pressure. (**A**) **i**: immunoprecipitation analysis of conjugated trans-acting factors (ITAFs) for RRBP1 and XIAP. Samples with (+) or without (−) reverse transcription (r.t.) were exhibited in different lanes; **ii**: Levels of La bound to RRBP1 bicistronic transcripts were quantified by qPCR and normalized to RRBP1 input levels. The FL/Input ratio from each cell line was compared with the ratio from wild-type Bel7402 cells, which was set to 1 (***: *p* < 0.0005); (**B**) Bel7402 cells were treated with PTBP1 siRNA, La siRNA or control, (non-targeting siRNA). Cell lysates were harvested and subjected to Western blot analysis. Bicistronic DNA constructs containing RRBP1 and XIAP IRES elements were transfected into cells after treatment with PTBP1 siRNA, La siRNA, or control siRNA, respectively, and then IRES activities were determined. The expression data are presented as the mean ± SEM of triplicate samples (**: *p* < 0.005); and (**C**) Western blot analysis of La expression levels in Bel7402 cells treated with 0.01, 0.2, and 0.4 μg/mL PTX or ADM for 12 h. During serum starvation, Bel7402 cells were growing in medium with 7.5% or 5% FBS for 12 h. Then the cell extracts were tested for both nuclear (Nuc) and cytoplasm (Cyt) expression of La and the total La in the whole cell extracts (WCE). The protein expression for La was normalized to β-actin and the resulting La/β-actin ratio from each treated cells was compared with the ratio from untreated cells, which was set to 1 (*: *p* < 0.05; ***: *p* < 0.0005).

To investigate the molecular mechanism of IRES-mediated translation, the mRNA-binding proteins of RRBP1 5′ UTR were determined. Previous studies have reported that ITAFs including PTBP1 and La were the binding protein of XIAP mRNA in vivo [26,27]. Therefore, the interaction of La and PTBP1 with RRBP1 was analyzed in Bel7402 cells with or without cellular stress conditions by RNA immunoprecipitation (RIP). The transfected Bel7402 cell lysates were immunoprecipitated with magnetic beads conjugated to La, PTBP1 antibody, or unconjugated beads (control). Total mRNA obtained from Bel7402 cell lysates (input) and immunoprecipitation (IP) with anti-La conjugated beads (La), anti-PTBP1 conjugated beads (PTBP1), or unconjugated beads (beads) were analyzed by reverse transcription polymerase chain reaction (RT-PCR) with FL primers. DNA contaminations were excluded by a control reaction without reverse transcription (r.t.). As shown in Figure 4Ai, both La and PTBP1 proteins were the binding protein of XIAP IRES, which was the same as previous reports. However, RRBP1 5′ UTR selectively bound La in wild-type (WT) cells, as well as in PTX-treated and serum-starved cells. Furthermore, quantitative real time polymerase chain reaction (qRCR) was performed to quantify the amount of RRBP1 bicistronic transcripts that interact with La in each cell line (Figure 4Aii). We found that there was more binding of La protein to the RRBP1 5′ UTR in PTX-treated and serum-starved cells than in WT cells, and this would agree with the higher activity of RRBP1 IRES that was observed in Bel7402 cells during stress conditions. These data would suggest that La is an activator of RRBP1 IRES in vivo. To further evaluate whether La regulates RRBP1 IRES-mediated translation, we performed gene transfection followed by reporter assay to examine both XIAP and RRBP1 IRES activities under conditions where PTBP1 or La is silenced by siRNA. We observed a reduced activity of XIAP IRES in those PTBP1 siRNA-transfected Bel7402 cells (Figure 4B). We also found that co-transfection of the La siRNA with pR-RRBP1-F reporter plasmid into Bel702 cells dramatically decreased the activity of RRBP1 IRES. 

After analyzing endogenous La protein by Western blot (WB), the increased dose of PTX or ADM resulted in a raise of La protein expression level in Bel7402 cells (Figure 4C), which possessed the same trend of RRBP1 5′ UTR IRES activity under the same drug pressure (Figure 3B). Moreover, PTX was more effective in enhancing the endogenous La production than ADM, which was the same effect when it was compared to RRBP1 IRES activity. During serum-starved conditions, the cytoplasm La expression was slightly increased in Bel7402 cells cultured with 7.5% FBS compared to that in normal cells. However, as expression of nuclear La was decreased in Bel7402 cells growing at 5% FBS, the cytoplasm La was significantly increased, suggesting that severe serum-starvation induces translocation of the La protein from the nucleus to the cytoplasm. Thus, the cytoplasm La protein can directly bind to RRBP1 IRES and regulate its activity. Therefore, the mechanism of IRES-mediated translation of RRBP1 was proposed that cellular stress induced RRBP1 IRES activity by increasing endogenous LA expression level in cytoplasm.

### 2.5. Mapping the RRBP1 IRES 

**Figure 5:**
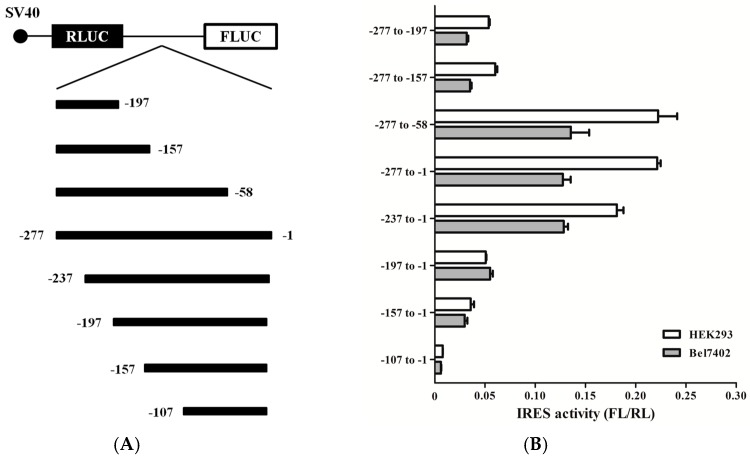

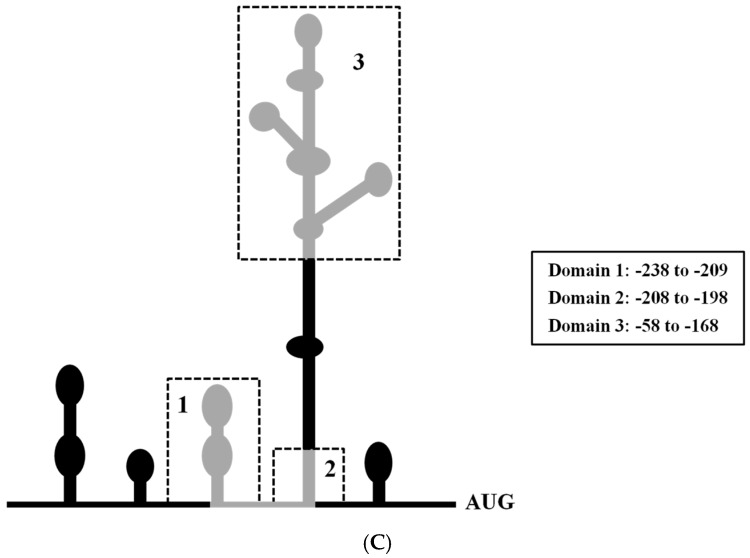
Mapping the RRBP1 IRES. (**A**) Schematic representation of the 5′- and 3’-deleted regions of the RRBP1 5′ UTR and these regions were inserted into pRF between Renilla and firefly luciferase reporter genes; (**B**) IRES activity of different truncated RRBP1 sequences in HEK293 and Bel7402 cell lines. The expression data are presented as the mean ± SEM of triplicate samples; and (**C**) schematic representation of the RRBP1 IRES, organized in domain 1–3 (grey line).

To determine the minimal sequences necessary for IRES activity, a series of bicistronic vectors were generated containing deletions of different size in the 5′-end or 3′-end of RRBP1 5′ UTR (Figure 5A). The resulting vectors were transfected into HEK293 and Bel7402 cells and luciferase activity was determined for each of these constructs. As seen from Figure 5B, IRES activities of truncated RRBP1 5′ UTR depended on the position in the 5′ UTR sequence. In all truncated RRBP1 5′ UTR, three sequences, -277 to -58, -277 to -1, and -237 to -1, had the similar (highest) IRES activity, indicating that the region between nucleotides -237 and -58 is required for maximal IRES activity. Further deletion of sequence between -237 and -58 resulted in dramatically decrease of IRES activity, such as -197, -157, and -107 to -1. 

Moreover, the position-dependent activity exhibited in two cell lines did not rely on the primary sequence but the secondary structure of RRBP1 IRES. The secondary structure of RRBP1 IRES element was folded by using Mfold software and the diagram of the structural motif was presented in Figure 5C. The internal deletion of 80 nt (-237 to -197) resulted in a 3.6-fold/2.3-fold (HEK293/Bel7402) decrease of IRES activity of the region between -237 and -1, accordingly. This important segment was organized in two modular domains. Domain 1 consists of two hairpin structures (-238 to -209) and domain 2, including single-strand and GC-rich sequences (-208 to -198). In addition, IRES activity decreases 3.7-fold/3.9-fold when 100 nt (-157 to -58) are deleted from the strong IRES element (-277 to -58) and this 100 nt of RRBP1 5′ UTR was organized in domain 3. Domain 3 consists of three structural motifs: two smaller stem-loops in different directions and a larger stem-loop with four hairpins (-58 to -168). We suggest that domains 1–3 may be necessary for ITAFs binding and the proteins that bind to these domains are important determinants of IRES activity, rather than the overall structure of the IRES itself.

### 2.6. Mutational Analysis of RRBP1 IRES

**Figure 6:**
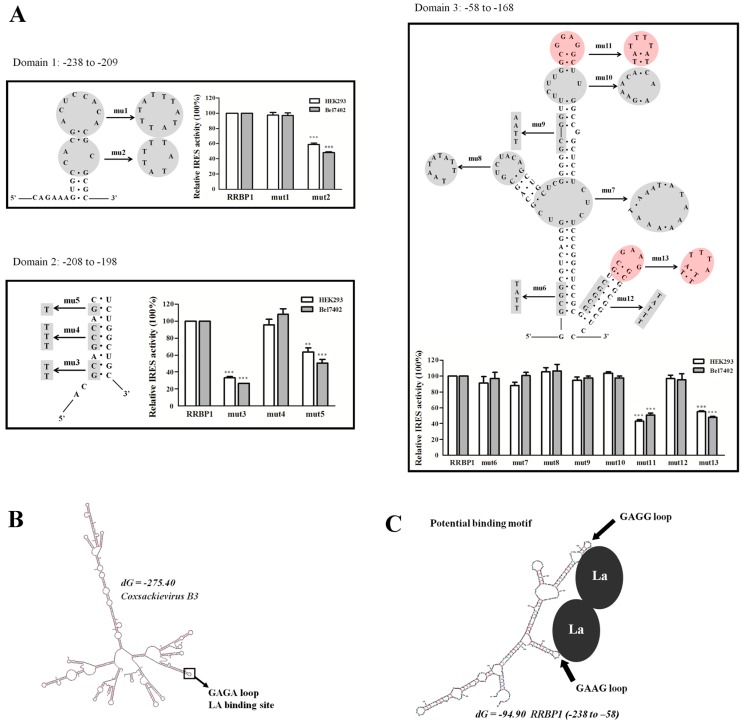
Mutational analysis of RRBP1 5′ UTR. (**A**) Thirteen mutations (with grey or red marker) which were made within the domains 1–3 of RRBP1 5′ UTR were inserted into the vector pRF, respectively. HEK293 and Bel7402 cells were transfected with either wild-type or the mutated versions of RRBP1 5′ UTR. The IRES activity from each cell line was determined and normalized to wild-type IRES (RRBP1, set to 100%). The expression data are presented as the mean ± SEM of triplicate samples (**: *p* < 0.005; ***: *p* < 0.0005); (**B**) the predicted secondary structure of coxsackievirus B3 (CVB3) 5′ UTR was generated by Mfold software indicating the position of the apical GAGA loop; and (**C**) the predicted secondary structure of RRBP1 IRES (-238 to -58) indicating the potential LA binding position of two apical GARR loops.

According to the simulated secondary structure and truncated sequences analysis, 13 mutants were generated for RRBP1 5′ UTR mutational analysis (named mut1–13). Each mutation site and sequence of RRBP1 5′ UTR was shown in Figure 6A. We focused on mutating typical structures on the stem loops which might cause partial disruption of the secondary structure of RRBP1 IRES. The relative IRES activities of the constructs mut1–3 were determined in HEK293- and Bel7402-transfected cells.

We observed that the activity of RRBP1 IRES decrease over 40% when a GC-rich (86%) loop was mutated at the domain 1 (Figure 6A, mut2) compared to the wild-type IRES, suggesting that the stability of this hairpin could be related with the efficiency of internal initiation of translation. Further mutation including mut3 and mut5 within the domain 2 of RRBP1 5′ UTR also remarkably decreased the IRES activity. According to the RNA structure model, these nucleotides are base-paired close to the GC-rich loop at domain 1 and may contribute to the tertiary structure required for ribosome recruitment. 

In addition, mutational analysis of domain 3 showed that two similar apical GA loops (with red markers) were necessary for RRBP1 IRES activity. It has been reported that coxsackievirus B3 (CBV3) RNA could bind La at an apical GAGA loop and influence internal initiation of translation [28,29] (Figure 6B). Therefore, we proposed that La may contact points close to the two similar GAGG and GAGG loops and facilitate ribosome recruitment (Figure 6C).

## 3. Discussion

Under stress conditions, such as serum starvation or hypoxia, translation of capped eukaryotic cellular mRNAs was downregulated and, meanwhile, the IRES elements act as a “fail-safe mechanism” to promote translation [30]. Moreover, several transcription factors, including p53, c-myc, and HIF-1α, were identified as IRES elements [31,32]. IRES translation pathway played an important role in maintaining stable expression level of housekeeping genes. Similar to these IRES elements, RRBP1, was crucial for transportation and secretion of intracellular proteins in mammalian cells. Meanwhile, it has been reported that overexpression of RRBP1 protein reduced ER stress and enhanced cell survival in some tumor cell lines [19,20]. The unusually long 5′ UTR of the RRBP1 protein may form an extensive secondary structure, which possesses IRES activity and, therefore, had several positive effects on tumor cells. In this study, the dual-luciferase plasmid containing RRBP1 mRNA was transfected into several cells and high IRES activity in cancer cells was detected. The difference of ITAFs may be the reason for different IRES activities in various cell lines. Furthermore, we tested the relative activity of RRBP1 5′ UTR with an additional dual-fluorescent protein reporter system. Although low fluorescence intensity of DsRed compared with EGFP [33], obvious expression of DsRed translated by RRBP1 5′ UTR was detected. Generally, the IRES from virus were much stronger than cellular IRES [27]. Similarly, we found the EMCV IRES was more efficient than RRBP1 IRES, indicating that RRBP1 IRES may be simulated during cell stress when cap-dependent translation is inhibited. 

During tumorigenesis, adaptive stress response caused the unfolded protein response (UPR) and initiated the activation of survival cascade mechanisms. Many previous studies reported that the development of tumorigenesis was influenced by the expression level of RRBP1, which upregulated the expression of UPR regulator protein BiP/GRP78 (binding immunoglobulin protein/glucose-regulated protein 78) [21]. Moreover, GRP78 resisted against ADM-mediated apoptosis in cancer cells, indicating RRBP1 overexpression cells possessed the resistance against ADM. Here, significant enhancement of endogenous RRBP1 protein expression was detected in Bel7402 cells under PTX or ADM condition without the increase of RRBP1 mRNA expression. During serum starvation, RRBP1 protein levels were also increased, which had a correlation with the simulated IRES activity. In addition, PTX or ADM also promoted the activity of RRBP1 5′ UTR. Therefore, the IRES activity of 5′ UTR of RRBP1 mRNA mediated the enhanced RRBP1 protein expression and made Bel7402 cells survive under cellular stress. 

The La is a member of RNA-binding proteins (RBPs) and play important roles in various cellular processes [34,35]. Within the nucleus, La associates with RNA polymerase III transcription and processing of tRNA precursors [36]. Within the cytoplasm, La binds to the 5′ UTR of several viral and cellular mRNA and simulates their translation in vivo [26,37,38]. In this study, ITAFs for XIAP IRES sequence were La and PTBP1, and for RRBP1 IRES sequence was La. The amount of La bound to RRBP1 IRES is significantly elevated in PTX-treated and serum-starved Bel7402 cells. Moreover, the expression of La was significantly enhanced in Bel7402 cells after treating with ADM or PTX. Although La is found originally in the nucleus of the cells, the La protein has been reported to shuttle between the nucleus and cytoplasm [39]. Here, we found severe serum starvation can induce cytoplasm translocation of La instead of affecting the total cellular La expression. These results indicated that IRES-mediated RRBP1 translation was regulator by La, and the enhancement of La protein in cytoplasm may be an important reason of different stress condition inducing RRBP1 expression in Bel7402 cells.

To detect the core region, IRES activity of truncated RRBP1 5′ UTR was analyzed and the region of 58–237 nt from the translation start site was essential for IRES activity. The diverse mechanisms used by IRESs in response to different cellular stress are reflected in their structural diversity [40]. Therefore, it is very important to characterize their RNA structure. For RRBP1 IRES, the predicted secondary structure of the functional region consists of three domains 1–3. We found the CCACGCG hairpin in domain 1, GC and G nucleotides in domain 2, and two small distal hairpins in domain 3 were indispensable for IRES activity. These results suggested that RRBP1 5′ UTR can be divided into sub-elements in nature that carry independent IRES activity, and these elements cooperate to the most efficient translation initiation in response to cellular stress. The GARR loop has been reported to be involved in interaction with the ITAFs, such as the apical GAGA loop binding LA protein [28,29]. However, mutation in this similar loop in domain 3 did not totally abrogate the IRES activity. This could be due to the fact that RRBP1 IRES also need other ITAFs to initiate translation which may bind the important GC-rich loop in domain 1. 

In this study, we demonstrate that RRBP1 5′ UTR harbor an IRES element which was independent on the choice of reporter genes. La stimulated the IRES activity of RRBP1 under stress conditions and the enhanced IRES activity, resulting in the increase of RRBP1 protein expression in Bel7402 cells, indicating the significant role of RRBP1 IRES response to cellular stress in hepatocellular carcinoma cells. Moreover, we established the potential secondary motif for LA protein binding by mutational analysis, which would help us elucidate the structure-function relation in RRBP1 IRES-mediated translation.

## 4. Experimental Section

### 4.1. Cell Lines, Cell Culture, Reagents, and Treatment

All cells were from the American Type Culture Collection. HEK293 and A2780 were cultured by Dulbecco’s modified Eagle’s Medium, where Bel7402 was maintained in Roswell Park memorial institute 1640 (RPMI 1640). All cells were cultured with 10% (*v*/*v*) fetal bovine serum (FBS) at 37 °C, 5% CO_2_. For drug treatment, Bel7402 cells were cultured in medium supplemented with clinically relevant concentration of PTX or ADM (Sigma, St. Louis, MO, USA), and without PTX or ADM (control) for 12 h. During serum starvation, Bel702 cells were grown in 1640 with 7.5% and 5% FBS for 12 h. 

### 4.2. Plasmid Construction

The detected 5′ UTR sequences in this study were synthesized by Sangon Biotech Co. (Shanghai, China) and were inserted into bicistronic plasmids between Renilla and firefly luciferase encoding genes. Bicistronic plasmids were constructed based on the pRF vector and has been described previously [41]. pBRF was constructed by deleting the SV40 promoter from pRF and used to analyze the cryptic promoter. Various sequences of 5′ UTR, including RRBP1, XIAP, and NRF, were inserted into plasmid pRF or pBRF between *Eco*R I and *Nde* I endonuclease restriction sites. To detect the core region for IRES activity, RRBP1 5′ UTR was deleted to a different length sequence by PCR and inserted into pRF. The production of the stem-loops mutants were also performed by PCR mutagenesis and some mutant products were created through two-step PCR by designing three primers and then inserted into pRF. The primer sequences used are described in the supplemental data (Tables S1 and S3). 

The dual-fluorescent protein reporter vector pGR is a bicistronic reporter with an enhanced green fluorescent protein gene and a red fluorescent protein gene. The RRBP1 and EMCV 5′ UTR were synthesized and inserted into vector pGR between *Xbal* I and *BstB* I endonuclease restriction sites to construct pG-RRBP1-G and pG-EMCV-G. The pBG-RRBP1-R vector without CMV promoter was created from a pBG-R vector, which was synthesized. The expression efficiency of the fluorescent protein was analyzed by fluorescence microscopy (Nikon, Tokyo, Japan).

### 4.3. Transient Transfection and Reporter Assay

All cells were transiently transfected by lipofectamine plus reagent (Invitrogen, Carlsbad, CA, USA) according to the protocol of the manufacturer. Renilla (RL) and firefly (FL) luciferase activities were detected by the dual-luciferase reporter assay system (Promega, Madison, WI, USA) according to the instruction of manufacturer. IRES activity was calculated as the average of FL/RL. Experiments were performed in triplicate.

### 4.4. Immunofluorescence

Bel7402 cells were plated in chamber slides, after growing for 24 h, cells were washed with phosphate buffer saline (PBS), and then fixed in 2% paraformaldehyde for 10 min at room temperature. The fixed cells were permeabilized with a blocking solution including 0.1% Triton X-100 and 5% bovine serum albumin in PBS for 30 min. Cells were then incubated with primary antibodies (anti-RRBP1 1:100) which were diluted in PBS containing 5% bovine serum albumin overnight at 4 °C. After washing three times with PBS, secondary antibody (Alexa Fluor 488 donkey anti-mouse 1:200 or Alexa Fluor 568 donkey anti-rabbit 1:200) diluted in PBS was added and incubated for 1 h at room temperature. Cells were washed in PBS and mounted in 4′,6-diamidino-2-phenylindole (DAPI) to counterstain DNA and observed by a confocal microscope (Leica TCS SP8, Solms, Germany).

### 4.5. Reverse Transcriptase (RT)-PCR and Quantitative (q) PCR

Total RNA was extracted by Trizol reagent following the manufacturer’s protocol (Invitrogen) and reverse transcribed using a PrimeScript^®^ 1st Strand cDNA Synthesis Kit (Takara, Dalian, China). qPCR was performed with SYBR^®^ Premix Ex Taq™ (Takara) according to the manufacturer’s instructions and quantified with a Roach Lightcycler 480 (Roach, Basel, Switzerland). Amplification products were visualized on a 1% agarose gel staining by ethidium bromide under UV light and primers for RT-PCR and qRCR are shown in supplementary data (Table S2).

### 4.6. Protein Extraction and Western Blot Analysis

To prepare the whole cell protein samples, Bel7402 cells were washed by phosphate-buffered saline, lysed by 150 μL RIPA buffer for 10 min at 4 °C and centrifuged at 12,000× *g* for 10 min. To detect the cellular localization of La, both nuclear and cytoplasm fractions were isolated using the NE-PER kit (Thermo Scientific™, Rockford, IL, USA) according to the protocol of the manufacturer. The obtained protein extracts in supernatant were separated by 10% SDS–PAGE and transferred to PVDF membranes. Proteins in PVDF membrane were incubated with antibodies of rabbit monoclonal anti-RRBP1 (Abcam, Cambridge, UK), mouse monoclonal anti-La/SSB (Abcam), mouse monoclonal anti-PTBP1 (Abcam) anti-Nucleolin (Abcam), β-actin, and β-tublin (Beyotime, Shanghai, China). Then proteins were detected by a chemiluminescent detection system (Tanon, Shanghai, China) and quantified by ImageJ software (NIH, Bethesda, MD, USA).

### 4.7. RNA-Protein Immunoprecipitation

In vivo crosslinking and co-precipitation of RNA-protein complexes were performed as methods described previously [15]. Cross-linked RNA-protein complexes were immunoprecipiated using anti-La/SSB or anti-PTBP1 (Santa Cruz Biotechnology, Shanghai, China) antibodies of 5 μg. Following immunoprecipitation and crosslink reversal, RNA was extracted as described above. RT-PCR and qRT-PCR were performed after reverse transcription of RNA to cDNA. The partial coding sequences of FL and β-actin were PCR amplified from the resulting cDNA using primers listed in supplemental data (Table S2).

### 4.8. siRNA Transfection

PTBP1, LA targeting and duplex control siRNAs (Santa Cruz Biotechnology) were transfected in Bel7402 (5 × 10^5^) by using Lipofactmine RNAiMax (Invitrogen) according the manufacturer’s protocol. After 48 h, cells were cotransfected with bicistronic reporter plasimds (3 μg of plasmids and 10 nM siRNA) containing XIAP and RRBP1 IRES elements with lipofectamine 2000 (Invitrogen), reporter protein levels were assayed after 24 h.

### 4.9. Statistical Analysis

Each experiment was performed at least three times. All experiments data were analyzed using GraphPad Prism 5.0 (Inc. 7825 Fay Avenue, La Jolla, CA, USA). and presented as mean values ± SEM. Statistical analysis was performed using a *t*-test (*: *p* < 0.05; **: *p* < 0.005; ***: *p* < 0.0005).

## Supplementary Materials

Supplementary materials can be found at http://www.mdpi.com/1422-0067/17/7/1174/s1.
