# Supplementary material for: La Autoantigen Induces Ribosome Binding Protein 1 (RRBP1) Expression through Internal Ribosome Entry Site (IRES)-Mediated Translation during Cellular Stress Condition
Source: Int J Mol Sci. 2016 Jul 20;17(7):1174. doi: 10.3390/ijms17071174 (PMC4964545; doi:10.3390/ijms17071174)
Supplement: Supplementary file 1 [file ijms-17-01174-s001.pdf]

Supplementary Materials: La Autoantigen Induces Ribosome Binding Protein 1 (RRBP1) Expression through Internal Ribosome Entry Site (IRES)-Mediated Translation during Cellular Stress Condition

Wenqing Gao, Qi Li, Ruiyu Zhu and Jian Jin

Table S1. Primers for deletion analysis.

Vector Construct	Prime Sequences 5′–3′
−277 to −197	F1: GGAATTC*CATATG*CGGCGATCCCGGCGGAGG<br>R1: CG*GAATTC*CGCTGGCTGC GTGCGCGC
−277 to −157	F1: GGAATTC*CATATG*CGGCGATCCCGGCGGAGG<br>R2: CG*GAATTC*CTGACGCCGC CCGCCCC
−277 to −58	F1: GGAATTC*CATATG*CGGCGATCCCGGCGGAGG<br>R3: CG*GAATTC*AGGAGCCGCCGCCTTCGCAG
−237 to −1	F2: GGAATTC*CATATG*CAGAAAGTGCCACGACTCCACA<br>R4: CG*GAATTC*CCTGGCTTGCTTTCCTTTCACCTG
−197 to −1	F3: GGAATTC*CATATG*GAGCGGCCGGAGCGGAC<br>R4: CG*GAATTC*CCTGGCTTGCTTTCCTTTCACCTG
−157 to −1	F4: GGAATTC*CATATG*GGGTCGCAGCGTCTACAGCT<br>R4: CG*GAATTC*CCTGGCTTGCTTTCCTTTCACCTG
−107 to −1	F5: GGAATTC*CATATG*GCCGGCTCCTCTCTCCCG<br>R4: CG*GAATTC*CCTGGCTTGCTTTCCTTTCACCTG

All primers are given in 5′ to 3′ direction. Restriction sites (RE) are in italic.

Table S2. Primers for RT-PCR and qRCR.

Description	Prime Sequences 5′–3′
RRBP1	F1: TTCAACGAGGGCGAGGCCCAG<br>R1: CGTGCCTGCACAGCCGTGATCT
β-actin	F2: TGAAGTGTGACGTGGACATC<br>R2: GGAGGAGCAATGATCTTGAT
FL	F3: ATGGAAGATGCCAAAAACATTAAGAAGG<br>R3: ACGGGCATGAAGAACTGCAAG

Table S3. Primers for mutational analysis.

Mutants	Prime Sequences 5′–3′
mut1	F1: GGAATTC*CATATG*CGGCGATCCCGGCGGAGGGGGCCGTTCGCCAGCTCTGAGGCAGAAA<br>GTGCCACTTTATTTATAGCGCGCACGCAGCCAGCGAGCGGCCGGAGCGGACGGCA
	R1: CG*GAATTC*CCTGGCTTGCTTTCCTTTCACC
mut2	F2: GGAATTC*CATATG*CGGCGATCCCGGCGGAGGGGGCCGTTCGCCAGCTCTGAGGCAGAAA<br>GTGATTTGACTCCACACTATCGCACGCAGCCAGCGAGCGGCCGGAGCGGACGGCA
	R1: CG*GAATTC*CCTGGCTTGCTTTCCTTTCACC
mut3	F3: GGAATTC*CATATG*CGGCGATCCCGGCGGAGGGGGCCGTTCGCCAGCTCTGAGGCAGAAA<br>GTGCCACGACTCCACACGCGCGCACTTAGCCAGCGAGCGGCCGGAGCGGACGGCA
	R1: CG*GAATTC*CCTGGCTTGCTTTCCTTTCACC
mut4	F4: GGAATTC*CATATG*CGGCGATCCCGGCGGAGGGGGCCGTTCGCCAGCTCTGAGGCAGAAA<br>GTGCCACGACTCCACACGCGCGCACGCATTTAGCGAGCGGCCGGAGCGGACGGCAGA
	R1: CG*GAATTC*CCTGGCTTGCTTTCCTTTCACC
mut5	F5: GGAATTC*CATATG*CGGCGATCCCGGCGGAGGGGGCCGTTCGCCAGCTCTGAGGCAGAAA<br>GTGCCACGACTCCACACGCGCGCACGCAGCCATCGAGCGGCCGGAGCGGACGGCAGA
	R1: CG*GAATTC*CCTGGCTTGCTTTCCTTTCACC
mut6	F6: GGAATTC*CATATG*CGGCGATCCCGGCGGAGGGGGCCGTTCGCCAGCTCTGAGGCAGAAA<br>GTGCCACGACTCCACACGCGCGCACGCAGCCAGCGA
	F7: CTCCACACGCGCGCACGCAGCCAGCGAGCGGCCGGAGCGGACGGCAGACGGGGCGGTT<br>ATCGTCAGGGTCGCAGCGTCTACA
	R1: CG*GAATTC*CCTGGCTTGCTTTCCTTTCACC
mut8	F8: GGAATTC*CATATG*CGGCGATCCCGGCGGAGGGGGCCGTTCGCCAGCTCTGAGGCAGAAA<br>GTGCCACGACTCCACACGCGCGCACGCAGCCAGCGAGCGGCCGGA
	F9: CGCGCGCACGCAGCCAGCGAGCGGCCGGAGCGGACGGCAGACGGGGCGGGCGGCGTC<br>AGGGTCGCAGTAATATATTCTGCTCGGGGGCGGTTTCTTGGCGGA
	R1: CG*GAATTC*CCTGGCTTGCTTTCCTTTCACC
mut9	F10: GGAATT*CCATATG*CGGCGATCCCGGCGGAGGGG
	R2: AGGAGCCGCCGCCTTCGCAGCCGCCGCGGAGCCGGGAGAGAGGAGCCGGCCAAGCCTC<br>CGCCAAGAAATTAACCCCGAGCAGCTGTAGACGCTGCGA
	R3: CG*GAATTC*CCTGGCTTGCTTTCCTTTCACCTGCAGCCAGAGACGCGAGAGGGAAAGCGAG<br>AGGGCAGGAGCCGCCGCCTTCGCAGCCGCCGCGGA
mut10	F10: GGAATT*CCATATG*CGGCGATCCCGGCGGAGGGG
	R4: GCCGCCGCCTTCGCAGCCGCCGCGGAGCCGGGAGAGAGGAGCCGGCTTGGCCTCCGCTG<br>TTTCACCGCCCCCGAGCAGCTGTAGACGCT
	R5: CG*GAATTC*CCTGGCTTGCTTTCCTTTCACCTGCAGCCAGAGACGCGAGAGGGAAAGCGAG<br>AGGGCAGGAGCCGCCGCCTTCGCAGCCGCCGCGGA
mut11	F10: GGAATT*CCATATG*CGGCGATCCCGGCGGAGGGG
	R6: GCCGCCGCCTTCGCAGCCGCCGCGGAGCCGGGAGAGAGGAGCCGGCCAAATAAAATAC<br>AAGAAACCGCCCCCGAGCAGCTGTAGA
	R7: CG*GAATTC*CCTGGCTTGCTTTCCTTTCACCTGCAGCCAGAGACGCGAGAGGGAAAGCGAG<br>AGGGCAGGAGCCGCCGCCTTCGCAGCCGCCGCGGA
mut12	F10: GGAATT*CCATATG*CGGCGATCCCGGCGGAGGGG
	R8: CG*GAATTC*CCTGGCTTGCTTTCCTTTCACCTGCAGCCAGAGACGCGAGAGGGAAAGCGAG<br>AGGGCAGGAGCCGCCGCCTTCGCAGCATAAACGGAGCCGGGAGAGAGGAGCCGGCCA
mut13	F10: GGAATT*CCATATG*CGGCGATCCCGGCGGAGGGG
	R9: CG*GAATTC*CCTGGCTTGCTTTCCTTTCACCTGCAGCCAGAGACGCGAGAGGGAAAGCGAG<br>AGGGCAGGAGCCGCCAATAAATAAGCCGCCGCGGAGCCGGGAGAGAGGA

All primers are given in 5′ to 3′ direction. Restriction sites (RE) are in italic. The sequence of mut8 was synthesized by Sangon Biotech. Co (Shanghai, China).
